# Disturbances in Switching between Canonical and Non-Canonical Wnt Signaling Characterize Developing and Postnatal Kidneys of *Dab1^−/−^* (*yotari*) Mice

**DOI:** 10.3390/biomedicines11051321

**Published:** 2023-04-28

**Authors:** Ilija Perutina, Nela Kelam, Mirko Maglica, Anita Racetin, Marin Ogorevc, Natalija Filipović, Yu Katsuyama, Josip Mišković, Katarina Vukojević

**Affiliations:** 1 Department of Anatomy, School of Medicine, University of Mostar, 88000 Mostar, Bosnia and Herzegovina; 2 Department of Anatomy, Histology and Embryology, University of Split School of Medicine, 21000 Split, Croatia; 3 Department of Medical Genetics, School of Medicine, University of Mostar, 88000 Mostar, Bosnia and Herzegovina; 4 Department of Anatomy, Shiga University of Medical Science, Otsu 520-2192, Japan; 5 Center for Translational Research in Biomedicine, University of Split School of Medicine, 21000 Split, Croatia

**Keywords:** canonical Wnt signaling, nephrogenesis, α-tubulin, inversin, dishevelled-1, Wnt5a/b, *β*-catenin, CAKUT, Dab1

## Abstract

This study aims to determine the protein expression patterns of acetylated α-tubulin, inversin, dishevelled-1, Wnt5a/b, and *β*-catenin in developing (E13.5 and E15.5) and early postnatal (P4 and P14) kidneys of *Dab1^−/−^* (*yotari*) mice, their role in regulating the Wnt signaling pathway, and the possible relation to congenital anomalies of kidney and urinary tract (CAKUT). The analysis of target protein co-expression, observed in the renal vesicles/immature glomeruli, ampullae/collecting ducts, convoluted tubules, metanephric mesenchyme of developing kidneys, but proximal convoluted tubules, distal convoluted tubules and glomeruli of postnatal kidneys, was performed using double immunofluorescence and semi-quantitative methods. The overall expression of acetylated α-tubulin and inversin during normal kidney development increases with higher expression in *yotari* mice as the kidney acquires mature morphology. An increase in *β*-catenin and cytosolic DVL-1 levels, indicating a switch from non-canonical to canonical Wnt signaling, is found in the postnatal kidney of *yotari* mice. In contrast, healthy mouse kidney expresses inversin and Wnt5a/b in the postnatal period, thus activating non-canonical Wnt signaling. Target protein expression patterns in kidney development and the early postnatal period observed in this study could indicate that switching between canonical and non-canonical Wnt signaling is crucial for normal nephrogenesis, while the defective *Dab1* gene product in *yotari* mice may promote CAKUT due to interfering with this process.

## 1. Introduction

The development of the metanephric kidney starts with two types of tubulogenesis. Initially, the ureteric bud (UB) branches off the mesonephric duct, invades the overlying metanephric mesenchyme (MM), and undergoes branching morphogenesis [1,2]. This induces the MM to condense at each UB branch tip and undergo a mesenchymal-to-epithelial transition (MET), forming the second set of tubules [3]. The UB is the basis for the development of the ureter, renal pelvis, calyces, and collecting ducts, while the MM is the basis for the development of nephrons, which pass through the renal vesicle, comma-shaped, and S-shaped body stage before connecting to the UB and becoming mature [2,4,5]. Mutual signaling interactions between the MM and UB ensure proper kidney development during nephrogenesis. If proper metanephric kidney development is interrupted, congenital anomalies of the kidney and urinary tract (CAKUT) occur. CAKUT encompasses a clinically broad spectrum of malformations, such as renal agenesis, renal hypoplasia/dysplasia, ureteropelvic junction obstruction, or vesicoureteral reflux, and contributes to 23% of congenital disabilities and accounts for 40% to 50% of pediatric cases and 7% of adult cases of end-stage renal disease (ESRD) worldwide [6,7].

Disabled 1 (DAB1), an intracellular adapter protein found in mice, is essential for a signal transduction pathway that coordinates the formation of neural networks and neuronal positioning in the developing brain. During kidney development, the canonical reelin/DAB1 pathway is crucial for cell fate decisions and differentiation [8]. A homozygous *Dab1^−/−^* mutation causes CAKUT to be displayed as kidney hypoplasia [9]. Reelin binds to its receptors and induces DAB1 tyrosine phosphorylation, and then phosphorylated DAB1 plays a role in activating various signaling pathways, such as Wnt signaling, by recruiting a diverse group of proteins that contain SH2 domains [10].

Glycogen synthase kinase-3 (GSK-3), a component of the protein-degrading complex, includes Axin, adenomatous polyposis coli (APC), and casein kinase 1, which typically targets cytosolic *β*-catenin in the absence of Wnt ligands. The destruction complex is bound to the cell membrane and disabled by DVL when one of the nineteen Wnt ligands binds to the frizzled receptor and the LDL receptor-related protein 5/6 coreceptors, enabling *β*-catenin to avoid degradation and accumulate in the nucleus, thus activating canonical Wnt signaling [11]. Genes targeted by canonical Wnt are regulated by *β*-catenin, which is bound to the T cell factor/lymphoid enhancer factor (TCF/LEF) in the nucleus. This leads to complex impacts on cellular responses such as proliferation, survival, and differentiation [12]. The non-canonical Wnt pathway is mediated by intracellular calcium ions and c-Jun N-terminal kinase [13]. The Wnt/Ca^2+^ pathway is one of the non-canonical Wnt pathways which causes intracellular calcium to be released and triggers calcium-dependent processes. Another non-canonical pathway is the Wnt/planar cell polarity (PCP) pathway, which is responsible for creating cell polarity and altering the arrangement of the cytoskeleton, leading to cell migration. In addition, the activation of several other transcription factors has also been observed [14]. 

In recent years, primary cilia have been shown to have key roles in kidney pathophysiology [15]. For example, primary cilia defects were related to irreversible kidney damage, such as fibrosis caused by epithelial-to-mesenchymal transition (EMT) of kidney tubule cells [16,17]. Four key events occur in tubular EMT: loss of epithelial adhesion, cytoskeletal reorganization and de novo synthesis of α-SMA, disruption of the tubular basement membrane, and finally, enhanced cell migration and invasion of the interstitium [18]. In the kidney, primary cilia were observed in most of the analyzed structures, including the parietal layer of Bowman’s capsule, proximal and distal convoluted tubules, and the collecting ducts, except for in the intercalated cells. The loss of acetylation of α-tubulin, the basic component of this microtubule-based organelle in immortalized cells, triggers EMT, thus implying that acetylated α-tubulin is important in the stabilization of microtubules [19]. Additionally, mutations of the ciliary protein inversin, which inhibits the canonical *β*-catenin depended on Wnt pathway by targeting cytoplasmic dishevelled (Dsh or DVL-1) for degradation, lead to nephronophthisis type II, an autosomal recessive cystic kidney disease characterized by extensive renal cysts, situs inversus, and renal failure [20]. Inversin is, therefore, essential in kidney morphogenesis [21,22]. There is enough evidence to indicate that Wnt signaling not only regulates ciliogenesis but that ciliary-mediated inhibition of the canonical Wnt pathway through the regulation of DVL-1 and the proteasome also appears to occur [23,24]. Crosstalk between cilia and the non-canonical Wnt planar cell polarity pathway is determined by PCP participating in both cilia orientation and in another type of polarity along the apical-basal axis, resulting in basal body docking and cilia outgrowth. Conversely, evidence for the role of cilia in PCP comes from the discovery that loss of *Ift20*, which encodes intraflagellar transport protein-20, results in cilia loss and causes misorientation of cell division and cystic kidney disease in mice [25]. 

Mice with *Wnt5a* deletions display a range of CAKUT phenotypes, from duplex kidneys and hydronephrosis to unilateral or bilateral renal agenesis [26]. *Wnt5a* has a role in PCP regulation in mice, and disruption in the PCP pathway has been shown to result in kidney cyst formation [27]. *β*-catenin-mediated canonical signaling acts transiently to induce the mesenchyme, as the downregulation of *β*-catenin activity is essential for the transition to the fully epithelial state of the renal vesicle [28]. Wnt/*β*-catenin signaling acts on tubular epithelial cells by upregulating certain of its target genes, some of which are important regulators of the tubular EMT process in the context of CAKUT, including fibroblast-specific protein 1 (FSP-1), matrix metalloproteinase 7 (MMP7), Snail, and Twist [29]. Inhibiting Wnt/*β*-catenin signaling consistently reduces renal fibrotic lesions and ameliorates kidney injury in a variety of chronic kidney disease models, indicating that targeting this signaling pathway may be an effective therapeutic intervention [30]. Through the suppression of the kinase Glycogen Synthase Kinase 3 beta, TGF-*β* was discovered to enhance the EMT process by upregulating *β*-catenin and Wnt signaling. When compared to control cells, *Arl13b* and *Ift20*-knock down cells had higher levels of EMT triggered by TGF-*β*, which resulted in decreased cilia elongation and higher expression of EMT markers like fibronectin, α-SMA, and collagen III [16,31]. 

We previously showed a slight increase in the expression of α-tubulin and inversin and a decreased expression of DVL-1 in healthy postnatal human kidneys [20]. Additionally, we showed that a homozygous *disabled 1* (*Dab1^−/−^*) mutation results in renal hypoplasia (CAKUT spectrum) accompanied with the foot process effacement in the glomeruli and the loss of functional structures [9,32,33]. Therefore, in this study, we aimed to analyze the protein expression of α-tubulin, inversin, DVL-1, Wnt5a/b, and *β*-catenin in the developing and early postnatal *yotari Dab1^−/−^* and wild-type mouse kidneys to determine whether alterations in Wnt signaling are present and their possible relation with the CAKUT spectrum.

## 2. Materials and Methods

### 2.1. Ethics

The Shiga University of Medical Science’s Guidelines for the Care and Use of Laboratory Animals permitted the use of animals in this research. The University of Split School of Medicine’s Ethical Committee granted its approval for the study, which was conducted according to the Declaration of Helsinki’s criteria (UP/1-322-01/17-01/13; 525-10/0255-17-7; 13 October 2017).

### 2.2. Sample Collection

This experiment used homozygous *Dab1^−/−^ yotari* mutant mice characterized by an autosomal recessive mutation of the *Dab1* gene, displaying phenotypes such as tremors, an unsteady gait, and early death around the time of weaning [32,33,34]. *Yotari (yot)* and C57BL/6N wild-type mice were kept apart and housed in groups in typical polycarbonate cages with unrestricted food and water access in a temperature-controlled environment. (23 ± 2 °C). The photoperiod consisted of 12 h of artificial light and 12 h of dark. The following PCR primers were used for genotyping: *yotari*—GCCCTTCAGCATCACCATGCT and CAGTGAGTACATATTGTGTGAGTTCC; wild type—GCCCTTCAGCATCACCATGCT and CCTTGTTTCTTTGCTTTAAGGCTGT [35]. To acquire their embryos, the gravid mice were sacrificed on embryonic days 13.5 (E13.5) and 15.5 (E15.5). On the fourth (P4) and fourteenth (P14) postnatal days, another group of mice was sacrificed. When the time point was examined, three mice of each genotype (*yotari* and wt) were analyzed. The mice were anesthetized by 1.5% sodium pentobarbital and transcardially perfused using phosphate buffer saline (PBS, pH 7.2) and 4% paraformaldehyde (PFA) in 0.1 M PBS [36]. Their kidneys were separately fixed with 4% PFA in 0.1 M PBS overnight for Hematoxylin-Eosin (HE) and immunofluorescence staining.

### 2.3. Immunofluorescence Staining

The tissue was first fixed and dehydrated using graded ethanol solutions (Sigma Aldrich in St. Louis, MO, USA) before being serially sliced into five µm-thick sections, embedded in paraffin blocks, and mounted on slides. The hematoxylin-eosin (HE) staining of every 10th section confirmed proper tissue preservation. The mounted tissue samples were heated in a sodium citrate buffer (Sigma Aldrich, St. Louis, MO, USA) for 20 min at 95 °C in a water steamer and then progressively cooled down to room temperature after deparaffinization in xylol and rehydration in graded ethanol and distilled water. After washing in 0.1 M PBS, protein-blocking buffer (ab64226, Abcam, Cambridge, UK) was applied for 20 min to prevent non-specific staining. Sections were consequently incubated in a humidity chamber overnight with primary antibodies (Table 1). The following day, they were rinsed with PBS before being incubated for an hour with the relevant secondary antibodies (Table 1). The samples were then rinsed in PBS thoroughly, and the nuclei were stained with DAPI and cover-slipped (Immuno-Mount, Thermo Shandon, Pittsburgh, PA, USA). The pre-adsorption test was completed so that each primary antibody was diluted in a blocking solution to the exact concentration. After adding an adequate peptide antigen, the sections were treated with the mixture. No non-specific secondary antibody binding or false-positive findings were observed when primary antibodies were omitted from the protocol.

### 2.4. Data Acquisition and Analysis

An immunofluorescent microscope (BX51, Olympus, Tokyo, Japan) equipped with a Nikon DS-Ri2 camera (Nikon Corporation, Tokyo, Japan) was used to analyze the sections. Non-overlapping visual fields were recorded at a magnification of x40 and constant exposure periods in order to determine the immunoexpression of proteins of interest. At least ten images of the embryonic kidney structures, namely, renal vesicles/immature glomeruli (Rv/G), ampullae/collecting ducts (A/Cd), convoluted tubules (Ct), and metanephric mesenchyme (MM), were taken at E13.5 and E15.5, but twenty images of the postnatal kidney structures, namely, glomeruli (G), proximal convoluted tubules (pct), and distal convoluted tubules (dct), were captured at P4 and P14. All analyzed images were processed with ImageJ software (NIH, Bethesda, MD, USA) and Adobe Photoshop (Adobe, San Jose, CA, USA) [37]. The co-expression of inversin and DVL-1, and Wnt5a/b and *β*-catenin were estimated by dividing the area of overlap using Adobe Photoshop. The quantity of positive cells in each group was averaged. Any level of nuclear, cytoplasmic, or membrane staining was considered positive. Different kidney structures’ staining intensity was semi-quantitatively assessed at four levels: the absence of any reactivity (−), mild reactivity (+), moderate reactivity (++), and strong reactivity (+++). Negative cells were categorized as cells with the absence of any immunoreactivity. Three researchers independently assessed the images while being blinded to the mouse strain and the time points. Interclass correlation analysis was used to examine the interrater agreement and produced a coefficient >0.75, suggesting excellent agreement [38].

### 2.5. Statistical Analyses

The statistical analysis was conducted using GraphPad Prism 9.0.0 software (GraphPad Software, San Diego, CA, USA). In order to identify significant variations in the percentage of positive cells, immunoexpression was compared using a two-way ANOVA test and Tukey’s multiple comparison test between Rv/G, A/Cd, Ct, and MM at E13.5 and E15.5 and G, pct, and dct at P4 and P14 of wild-type and *yotari* mice. The percentage of positive cells was represented as mean value standard deviation (SD), with *p* < 0.05 being the threshold for statistical significance.

## 3. Results

*Yotari* (*yot*) *Dab1^−/−^* and wild-type (wt) mouse kidneys were used to assess the protein expression patterns of acetylated α-tubulin, inversin, DVL-1, Wnt5a/b, and *β*-catenin markers in the glomeruli (G), proximal (pct), and distal convoluted tubules (dct) at postnatal days P4 and P14. Embryonic structures including renal vesicles/immature glomeruli (Rv/G), ampullae/collecting ducts (A/Cd), convoluted tubules (Ct), and metanephric mesenchyme (MM) were examined using mice at gestation days E13.5 and E15.5. Focus was particularly placed on the co-expression of inversin with DVL-1 and Wnt5a/b with *β*-catenin. The obtained markers showed positive expression patterns but with differences in intensity (semi-quantitative analysis described in Table 2), distribution, and quantity. 

### 3.1. Acetylated α-Tubulin Immunoexpression

**Figure 1:**
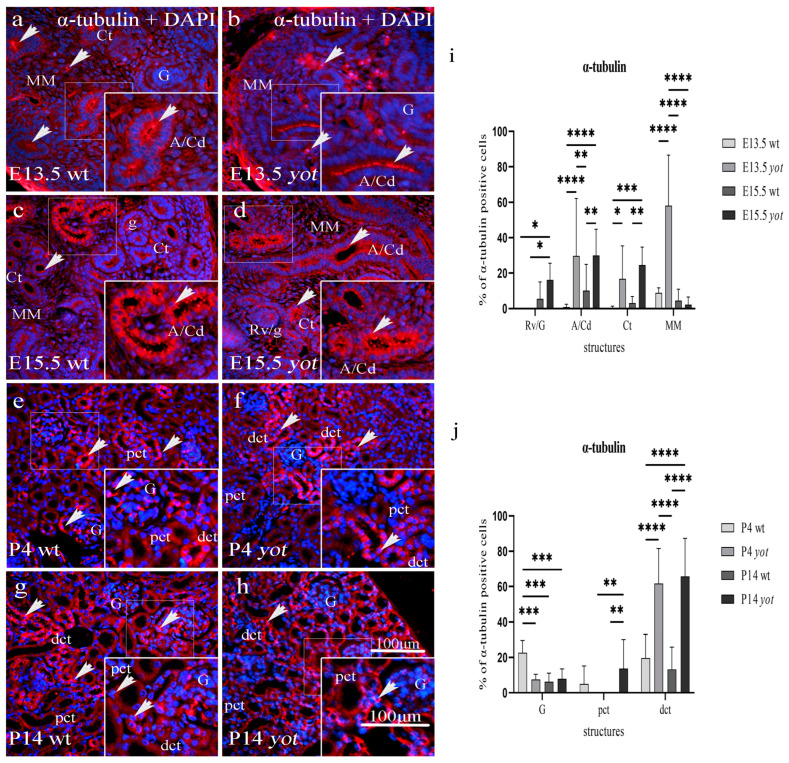
Immunofluorescence staining of acetylated α-tubulin merged with DAPI in the developing and postnatal wild-type (wt) and *yotari* (*yot*) kidneys (**a**–**h**). Acetylated α-tubulin distribution comparison between kidneys at embryonic days 13.5 (E13.5) and 15.5 (E15.5) (**a**–**d**), and postnatal days 4 (P4) and 14 (P14) of wt and *yot* mice (**e**–**h**). Positive staining of acetylated α-tubulin (arrows) is shown in each substructure in the kidney (**a**–**h**). Renal vesicles/immature glomeruli (Rv/G), ampullae/collecting ducts (A/Cd), convoluted tubules (Ct), and metanephric mesenchyme (MM) at E13.5 and E15.5 (**a**–**d**). Proximal convoluted tubules (pct), distal convoluted tubules (dct), and glomeruli (G) at P4 and P14 (**e**-**h**) of wt and *yot* mice. Magnification ×40, scale bar 100 µm. The percentage of acetylated α-tubulin positive cells in wt and *yot* mice per structure throughout different stages of developing (**i**) and postnatal kidney (**j**). Ten substructures were assessed at E13.5 and E15.5 and twenty at P4 and P14. Data are displayed as the mean ± SD (vertical line) and analyzed by a two-way ANOVA test followed by Tukey’s multiple comparison test. Significant differences are indicated by the following: * *p* < 0.05; ** *p* < 0.01; *** *p* < 0.001; **** *p* < 0.00001.

Acetylated α-tubulin was expressed in all developing and postnatal kidney structures of *yotari* and wild-type mice. Strong punctate protein expression was shown within the apical membrane of collecting ducts, including ampullae and convoluted tubules during E13.5 and E15.5, but only diffuse perinuclear cytoplasmatic expression was shown in the metanephric mesenchyme (interstitium) during E13.5, with a statistically significant increased expression in *yot* compared to wt mice (*p* < 0.05, Figure 1a–d,i, Table 2). Among different developmental stages, the strongest expression of acetylated α-tubulin protein was found in the MM of the E13.5 *yot* kidneys, containing 58% of positive cells, predominantly staining cells close to the collecting duct, while towards the periphery, the expression had a reverse pattern (Figure 1b). The expression in undifferentiated cells of the Rv/G stages was weak in both mouse types, but a greater expression was found in *yot* mice.

Acetylated α-tubulin was strongly expressed in the perinuclear cytoplasm and apical membrane of postnatal kidney structures, specifically the visceral epithelial cells (podocytes) of Bowman’s capsule and the dct; however, the pct showed weak protein expression in both mouse types (Figure 1e–h). The expression in the wt dct dropped from 19.55% of positive cells at P4 to 13.25% at P14, while it elevated from 61.64% to 65.75% during the same period in the *yot* dct (Figure 1j). Postnatal staining of *yot* dct was significantly increased compared to wt dct (*p* < 0.0001, Figure 1j). The strongest immunoreactivity to acetylated α-tubulin was observed on the apical cell surface and cytoplasm of *yotari* mice dct at P14, with 65.75% positive cells. In the P4 wt group, 22.65% of the glomerular cells expressed acetylated α-tubulin, significantly higher than the other three observed groups (*p* < 0.001).

A semi-quantitative analysis revealed the mild staining reactivity of acetylated α-tubulin in glomeruli but moderate in distal convoluted tubules of wild-type and *yotari* mice during the postnatal period (Table 3).

### 3.2. Double Immunofluorescence Staining to Inversin and DVL-1 in Developing and Postnatal Wild-Type and Yotari Mouse Kidneys

#### 3.2.1. Inversin and DVL-1 Expression in Developing Kidney of Wild-Type and Yotari Mice

**Figure 2:**
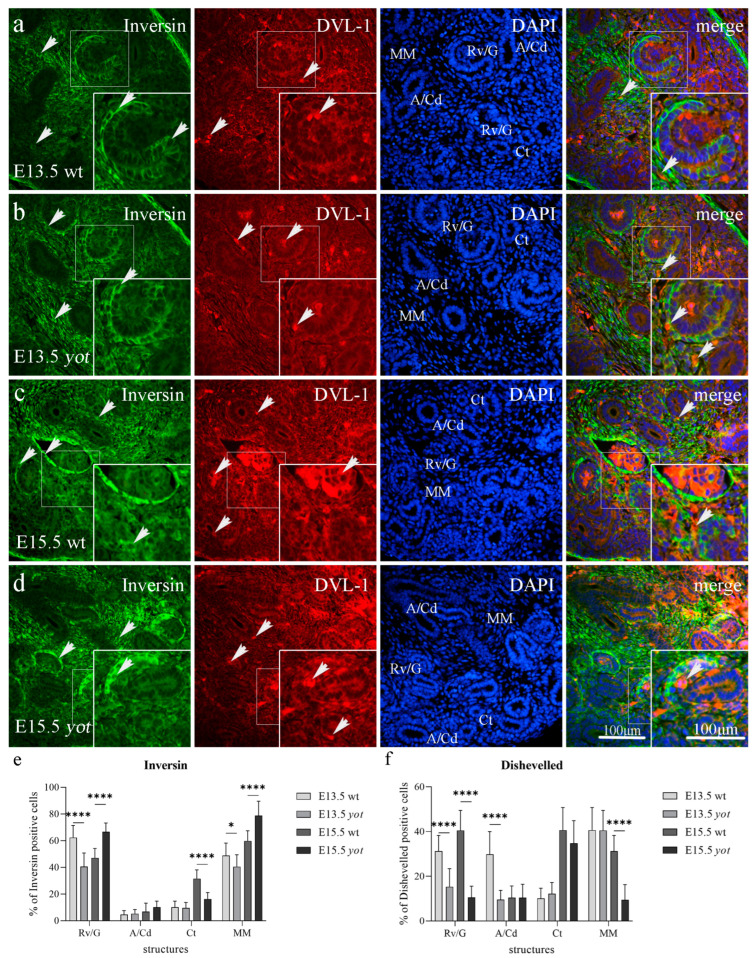
Double immunofluorescence staining of inversin (green), DVL-1 (red), and DAPI (blue) in the developing kidneys at embryonic days 13.5 (E13.5) and 15.5 (E15.5) of wild-type (wt) and *yotari* (*yot*) mice. Positive staining (arrows) is shown in each structure throughout all phases of development (**a**–**d**). Merged microphotographs along with structures of interest in the cortex: renal vesicles/immature glomeruli (Rv/G), ampullae/collecting ducts (A/Cd), convoluted tubules (Ct), and metanephric mesenchyme (MM) at E13.5 and E15.5. Co-expression of inversin/DVL-1 (arrowhead) is shown in merged microphotographs. Details are shown as higher magnification insets. Magnification ×40, scale bar 100 µm. Dynamics of positive cell distribution of inversin and DVL-1 in kidney structures (Rv/G, A/Cd, Ct, and MM) throughout developing stages are shown in graphs (**e**,**f**). Ten substructures were assessed at each time point. Data are displayed as the mean ± SD (vertical line) and analyzed by a two-way ANOVA test followed by Tukey’s multiple comparison test. Significant differences are indicated by the following: * *p* < 0.05; **** *p* < 0.00001.

During E13.5, inversin showed strong diffuse cytoplasmatic expression in the parietal layer of renal vesicles and the metanephric mesenchyme of wild-type and *yotari* mice. Strong DVL-1 expression was observed in the perinuclear cytoplasm in all wild-type and *yotari* embryonic kidney substructures (Figure 2a,b). Inversin immunoreactivity, dispersed in the cytoplasm of the wt mice parietal layer of renal vesicles and metanephric mesenchyme, was significantly increased when compared to *yotari* mice at the same developmental stage but decreased at E15.5 (*p* < 0.05). Ct showed greater expression in wild-type compared to *yotari* mice at E15.5 (*p* < 0.0001, Figure 2e). A higher expression of DVL-1 was observed in the renal vesicles and convoluted ducts of wild-type mice at E13.5 compared to *yotari* (*p* < 0.0001). Wild-type mice at E15.5 showed higher protein expression of DVL-1 compared to *yotari* in renal vesicles, convoluted tubules and metanephric mesenchyme (Figure 2f). A co-expression of inversin and DVL-1 was observed in the cytoplasm of all embryonic kidney structures during the development of wild-type and *yotari* mice (see Figure 2a–d, merge). 

We found expression of inversin in all investigated embryonic structures with a mean protein expression of 54.75% positive cells for Rv/G, 5.8% for A/Cd, 20.8% for Ct, and 54.35% for MM of the wild-type at day E13.5 and E15.5, but 53.6% for Rv/G, 7.7% for A/Cd, 12.95% for Ct, and 59.65% for MM of *yotari* mice at day E13.5 and E15.5. We observed the difference in signal strength of inversin and DVL-1 expression between structures, where moderate to strong diffuse cytoplasmatic inversin expression was observed in the parietal layer of renal vesicles and metanephric mesenchyme of wild-type and *yotari* mice, but only mild to moderate DVL-1 protein expression in the same structures during kidney development (Table 2). The highest expression of inversin was found in MM of *yotari* mice at E15.5 with a mean of 78.8% of positive cells. The mean expression of DVL-1 positive cells was 35.85% for Rv/G, 20.1% for A/Cd, 25.35% for Ct, and 35.9% for MM of the wild-type at day E13.5 and E15.5, but 12.95% for Rv/G, 10% for A/Cd, 23.45% for Ct, and 24.95% for MM of *yotari* mice at day E13.5 and E15.5. The highest expression of DVL-1 was found in the Ct of wt mice at E15.5 and the MM of wt mice at E13.5, both with a mean of 40.6% of positive cells. 

#### 3.2.2. Inversin and DVL-1 Expression in Postnatal Kidney of Wild-Type and Yotari Mice

**Figure 3:**
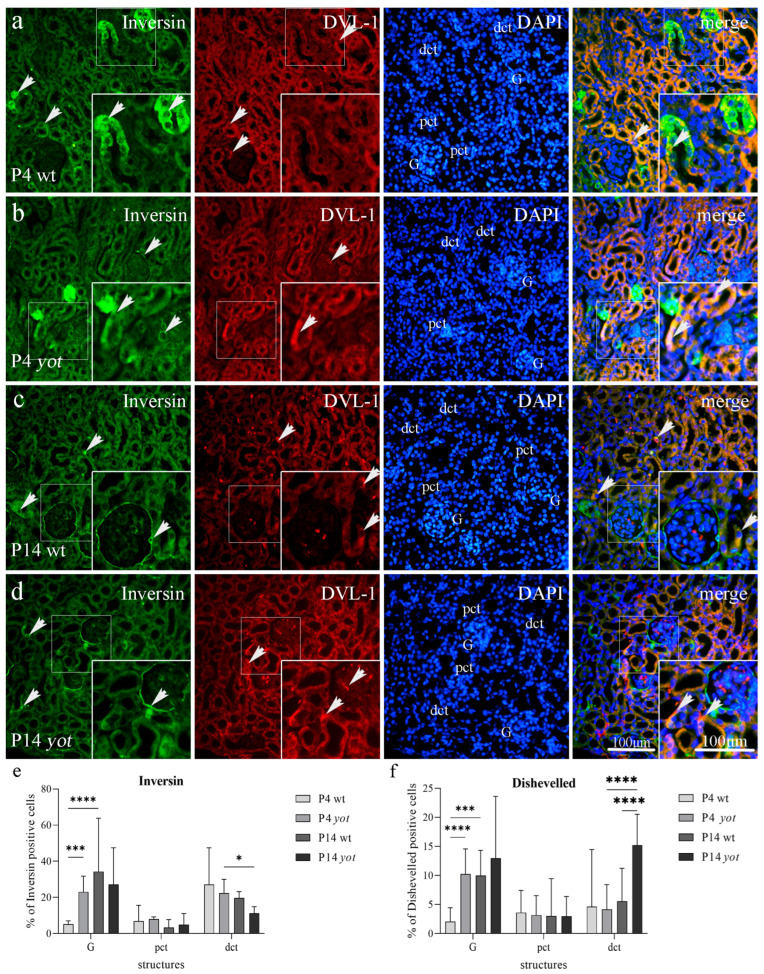
Double immunofluorescence staining of inversin (green), DVL-1 (red), and DAPI (blue) in the kidneys of wild-type (wt) and yotari (yot) mice at 4th (P4) and 14th postnatal day (P14). Positive staining (arrows) is shown in each structure throughout all phases of development (**a**–**d**). Merged microphotographs along with structures of interest in the cortex: proximal convoluted tubules (pct), distal convoluted tubules (dct), and glomeruli (G) at P4 and P14. Co-expression of inversin/DVL-1 (arrowhead) is shown in merged microphotographs. Details are shown as higher magnification insets. Magnification ×40, scale bar 100 µm. Dynamics of positive cell distribution of inversin and DVL-1 in kidney structures (G, pct, and dct) throughout postnatal stages are shown in graphs (**e**,**f**). Twenty substructures were assessed at each time point. Data are displayed as the mean ± SD (vertical line) and analyzed by a two-way ANOVA test followed by Tukey’s multiple comparison test. Significant differences are indicated by the following: * *p* < 0.05; *** *p* < 0.001; **** *p* < 0.0001.

Inversin demonstrated diffuse cytoplasmic staining in the parietal layer of G and dct, and weak reactivity in pct in the cortex of wild-type and *yotari* mouse kidneys at P4. DVL-1 is observed in the cytoplasm of G, pct, and dct on the same day in both mouse genotypes (Figure 3a,b, Table 3). *Yotari* mice at P4 demonstrated an increased expression of inversin protein compared to the wild-type in glomeruli (*p* < 0.001). During P14, inversin immunoreactivity was present in the cytoplasm of the parietal layer of Bowman’s capsule in wt mice. Inversin reactivity drops in dct as the kidney acquires mature morphology (Figure 3e). There was no statistically significant difference in inversin and DVL-1 positive cells in the pct of both genotypes in the postnatal period. DVL-1 presented positive expression in the perinuclear cytoplasm of all postnatal structures at P14. Wild-type mice at P4 day showed lower protein expression of DVL-1 in podocytes compared to *yotari* mice (*p* < 0.0001). DVL-1 expression in G and dct was elevated significantly from P4 to P14 in *yotari* mice (*p* < 0.001). Co-expression of inversin and DVL-1 characterized cellular compartments of tubular cells in the pct and dct (Figure 3a–d, merge). 

Inversin expression pattern was demonstrated in all investigated postnatal structures, including G, pct, and dct, with a mean protein expression of positive cells of 19.71% for G, 5.13% for pct, and 23.5% for dct of the wild-type at P4 and P14, but 25.12% for G, 6.48% for pct, and 16.84% for dct of *yotari* mice at day P4 and P14 (Figure 3e). The highest expression of inversin was found in G of wt P14, with a mean of 34.27% positive cells. The mean expression of DVL-1 positive cells was 6.02% for G, 3.31% for pct, and 5.1% for dct of the wild-type, but 11.62% for G, 3.07% for pct, and 9.69% for dct of *yotari* mice at P4 and P14. The highest expression of DVL-1 was found in the dct of wt P14 with a mean of 15.21% positive cells (Figure 3f). 

### 3.3. Double Immunofluorescence Staining to Wnt5a/b and β-Catenin in Developing and Postnatal Wild-Type and Yotari Mouse Kidneys

#### 3.3.1. Wnt5a/b and *β*-Catenin Expression in Developing Kidney of Wild-Type and Yotari Mice

**Figure 4:**
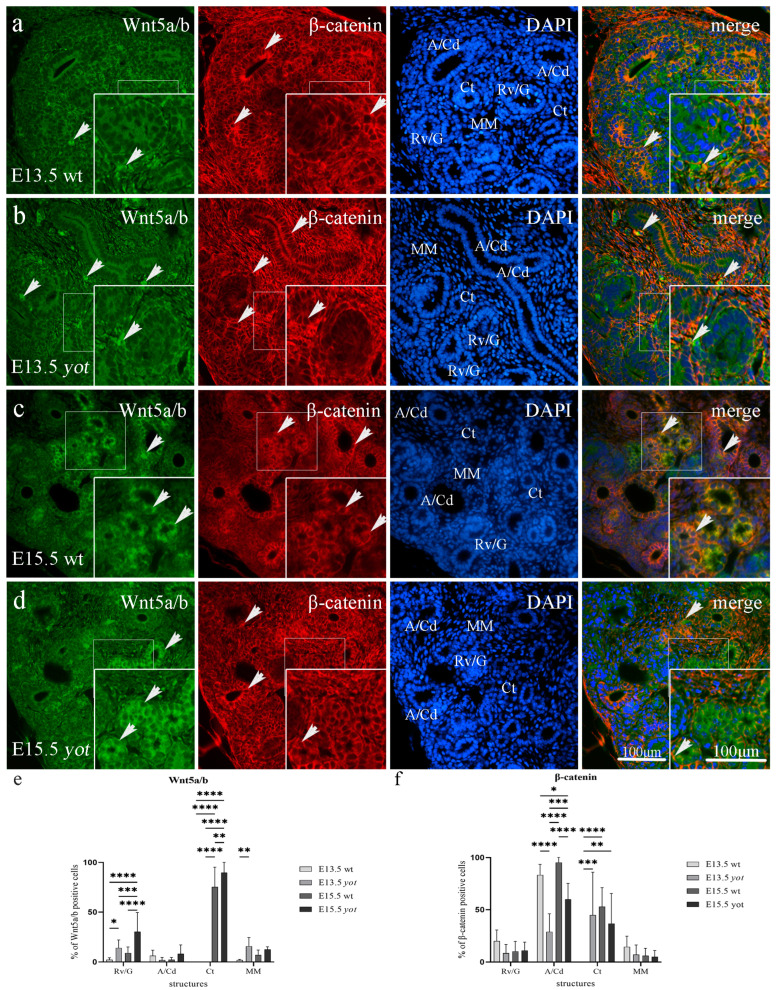
Double immunofluorescence staining of Wnt5a/b (green), *β*-catenin (red), and DAPI (blue) in the developing kidneys at embryonic days 13.5 (E13.5) and 15.5 (E15.5) of wild-type (wt) and *yotari* (*yot*) mice. Positive staining (arrows) is shown in each structure throughout all phases of development (**a**–**d**). Merged microphotographs along with structures of interest in the cortex: renal vesicles/immature glomeruli (Rv/G), ampullae/collecting ducts (A/Cd), convoluted tubules (Ct), and metanephric mesenchyme (MM) at E13.5 and E15.5. Co-expression of Wnt5a/b/*β*-catenin (arrowhead) is shown in merged microphotographs. Details are shown as higher magnification insets. Magnification ×40, scale bar 100 µm. Dynamics of positive cell distribution of Wnt5a/b and *β*-catenin in kidney structures (MM, Rv/G, A/Cd, and Ct) throughout developing stages are shown in graphs (**e**,**f**). Ten substructures were assessed at each time point. Data are presented as the mean ± SD (vertical line) and analyzed by a two-way ANOVA test followed by Tukey’s multiple comparison test. Significant differences are indicated by the following: * *p* < 0.05; ** *p* < 0.01; *** *p* < 0.001; **** *p* < 0.0001.

The expression of Wnt5a/b protein was mild to moderate in the perinuclear cytoplasm of developing structures, such as renal vesicles and metanephric mesenchyme, of *yotari* mice, and was increased compared to wild-type mouse kidneys at E13.5, while strong membranous expression of *β*-catenin was observed on the apical and basal membrane of all wild type and *yotari* embryonic structures (Rv/G, A/Cd, Ct, MM) (Figure 4a–d, Table 2). *Yotari* mice showed higher expression of Wnt5a/b in renal vesicles compared to wild-type mice, and additionally in convoluted tubules at E15.5 (*p* < 0.05). *β*-catenin immunofluorescence staining was significantly increased in the ampullae but decreased in the convoluted tubules of E13.5 wt compared to *yotari* (*p* < 0.001). During E15.5 *β*-catenin immunoreactivity was increased in wt collecting ducts and convoluted tubules when compared to *yotari* mice. The co-expression of Wnt5a/b and *β*-catenin was observed in the metanephric mesenchyme during the development of wild-type and *yotari* kidneys (see Figure 4a–d, merge). 

We found expression of Wnt5a/b protein in all investigated embryonic structures, including Rv/G, A/Cd, Ct, and MM, with a mean expression of positive cells of 5.62% for Rv/G, 4.37% for A/Cd, 37.74% for Ct, and 4.52% for MM of wild-type at day E13.5 and E15.5, but 22.18% for Rv/G, 5.08% for A/Cd, 44.91% for Ct, and 14.12% for MM for *yotari* at day E13.5 and E15.5 (Figure 4e). The highest protein expression of Wnt5a/b was found in the Ct of *yotari* E15.5, with a mean of 89.93% of positive cells. The mean expression of *β*-catenin positive cells was 1.58% for Rv/G, 86.93% for A/Cd, 22.19% for Ct, and 1.74% for MM of the wild type at day E13.5 and E15.5, but 9.7% for Rv/G, 23.64% for A/Cd, 7.39% for Ct, and 0.28% for MM of *yotari* at day E13.5 and E15.5. The highest expression of *β*-catenin was found in A/Cd of wt E15.5, with a mean of 95.39% of positive cells (Figure 4f). 

#### 3.3.2. Wnt5a/b and *β*-Catenin Expression in Postnatal Kidney of Wild-Type and Yotari Mice

**Figure 5:**
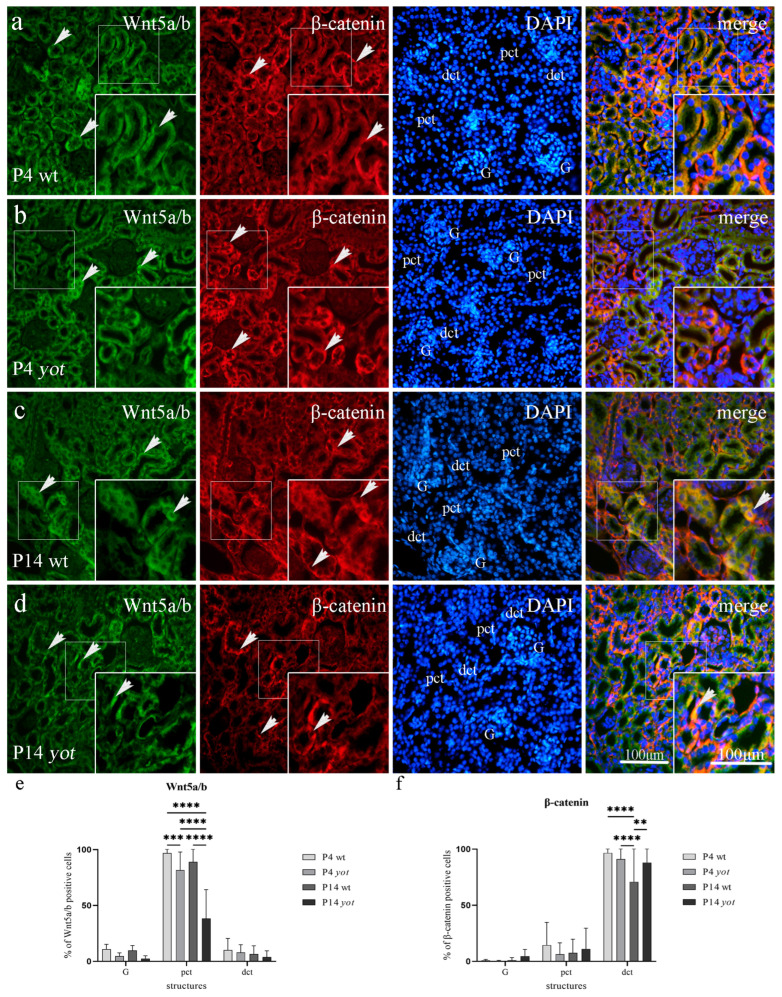
Double immunofluorescence staining of Wnt5a/b (green), *β*-catenin (red), and DAPI (blue) in the kidneys of wild-type (wt) and *yotari* (*yot*) mice at 4th (P4) and 14th postnatal day (P14). Positive staining (arrows) is shown in each structure throughout all phases of development (**a**–**d**). Merged microphotographs along with structures of interest in the cortex: proximal convoluted tubules (pct), distal convoluted tubules (dct), and glomeruli (G) at postnatal days P4 and P14. Co-expression of Wnt5a/b/*β*-catenin (arrowhead) is shown in merged microphotographs. Details are shown as higher magnification insets. Magnification ×40, scale bar 100 µm. Dynamics of positive cell distribution of Wnt5a/b and *β*-catenin in kidney structures (G, pct, and dct) throughout postnatal stages are shown in graphs (**e**,**f**). Twenty substructures were assessed at each time point. Data are displayed as the mean ± SD (vertical line) and analyzed by a two-way ANOVA test followed by Tukey’s multiple comparison test. Significant differences are indicated by the following: ** *p* < 0.01; *** *p* < 0.001; **** *p* < 0.0001.

Wnt5a/b showed strong diffuse cytoplasmatic staining in the visceral layer of G and dct, and the strongest reactivity in pct of postnatal wild-type and *yotari* mouse kidneys at P4. At the same time, *β*-catenin was present predominantly in the basal membrane of G and pct, with the strongest staining intensity in dct at P4 of both mouse types (Figure 5a,b, Table 3). Wild-type mice at P4 and P14 showed a statistically significant increased expression of Wnt5a/b protein compared to *yotari* mice in pct (*p* < 0.001). There was no statistically significant difference in Wnt5a/b expression in dct and G of wt and *yot* mice at P4 or P14, and the same was true for *β*-catenin expression in the G and pct of wt and *yot* mice (Figure 5e,f, Table 3). Wild-type mice at P14 showed a dropping expression of *β*-catenin protein in the dct compared to P4 (*p* < 0.0001). *β*-catenin of *yotari* P14 was also decreased in relation to P4 but remains statistically higher when compared to wild-type *β*-catenin at day P14 (*p* < 0.01, Figure 5c,d,f). The co-expression of Wnt5a/b and *β*-catenin was noticed only occasionally in pct and dct at day P14 (see Figure 5c,d, merge). 

We found expression of Wnt5a/b in all investigated postnatal structures, including G, pct, and dct, with a mean protein expression of positive cells of 10.34% for G, 92.93% for pct, and 8.4% for dct of wild-type mice at day P4 and P14, but 3.65% for G, 59.94% for pct, and 5.96% for dct of *yotari* mice at P4 and P14. The highest expression of Wnt5a/b was found in pct of wt P4 with a mean of 96.85% positive cells (Figure 5e). The mean expression of *β*-catenin positive cells was 0.03% for G, 0.37% for pct, and 95.3% for dct of the wild-type mice at P4 and P14, but of 0.06% for G, 3.48% for pct, and 91.04% for dct of *yotari* mice at P4 and P14. The highest expression of *β*-catenin was found in the dct of wt mice at P4 with a mean of 96.59% positive cells (Figure 5f).

## 4. Discussion

Nephrogenesis, extending even into the early postnatal period of mice, is composed of complex processes precisely coordinated by the interaction of a large number of genes, whereas CAKUT has been associated with mutations in more than 20 genes so far [6,39]. Due to the high expression of DAB1 in our prior research during normal human fetal kidney development, we hypothesized that DAB1 might be involved in kidney development [9]. The aim of this study, where we compared the *yotari* (*Dab1^−/−^*) mouse model to wild-type mice, was to determine the differences in spatio-temporal distribution patterns of acetylated α-tubulin, inversin, DVL-1, Wnt5a/b, and *β*-catenin in both embryonic and postnatal developmental stages and their correlation with the switch between canonical and non-canonical Wnt signaling, resulting with CAKUT when interrupted. 

Primary cilium-mediated Wnt pathway activation during kidney development enables cell proliferation, differentiation, and tissue morphogenesis. Many children with impaired primary cilia function and disrupted Wnt signaling develop chronic kidney disease (CKD) [40]. Primary cilia function as a switch between canonical and non-canonical Wnt signaling activity, possibly determining between regenerative and pro-fibrotic effects of Wnt re-expression in the injured kidney [41]. The study of Han et al. highlighted the significance of maintaining primary cilia during acute kidney injury (AKI), such as ischemia/reperfusion injury and kidney transplantation, in order to avoid pro-fibrotic kidney changes due to epithelial-to-mesenchymal transition [16]. Further studies are needed to determine the ultrastructural morphology of primary cilia and whether EMT is activated in the kidneys of *yotari* mice. 

The canonical Wnt/*β*-catenin signaling pathway regulates cell fate, proliferation, and survival during the early stages of kidney morphogenesis. Using *β*-catenin responsive TCF/*β*-gal reporter mic, canonical Wnt signaling activation was found in the epithelia of branching UB and induction during the progression of the MM to tubular differentiation [42,43]. In contrast, the non-canonical planar cell polarity Wnt pathway is more associated with differentiation, cell polarity, and migration, thus controlling the process of MET. Some non-canonical/PCP components are expressed in the renal developing epithelia, such as the UB, Rv, and S-shaped body, indicating their roles in cell division orientation, movements, adhesion, and contribution to morphogenesis of the mature nephron [44]. The conclusion that the canonical Wnt signaling pathway is more active in early nephrogenesis while it becomes silenced as the nephron acquires mature morphology and that the opposite is true for the non-canonical PCP pathway explains that the switch from canonical to non-canonical Wnt signaling is crucial for normal nephrogenesis [20,28]. Wnt/*β*-catenin is activated in animal models of both acute kidney injury and chronic kidney disease, which might function as a protective or harmful mechanism for the kidneys [45]. Our study shows the switch from non-canonical to canonical Wnt signaling in the postnatal kidney of *yotari* mice, which could lead to CAKUT. 

Results of this study determine that overall protein expression of acetylated α-tubulin and inversin during normal kidney development is increased, with higher expression in *yotari* mice as kidneys acquired mature morphology, except for acetylated α-tubulin in the MM where overall expression is decreased and inversin in the Ct where higher expression is obtained in wild-type mice. On the contrary, a study by Solic et al. showed decreasing overall expression of these markers as kidney structures mature [20]. DVL-1 protein expression elevates in Rv/G and Ct of wild-type mice during nephrogenesis but drops in A/Cd and MM, while it elevates in Cd and Ct and drops in Rv/G and MM of *yotari* mice, compared to the decreasing expression in our previous study. There was an occasional co-expression of the inversin and DVL-1 proteins, mostly in the MM during nephrogenesis, pct, and dct during the postnatal period. Similar to the study of Solic I et al., our results showed a slight increase in the expression of inversin but a decrease of DVL-1 in healthy postnatal kidneys that corroborates the conclusion that the non-canonical Wnt pathway remains active after birth, but the canonical Wnt pathway is silenced [20]. These findings support our earlier research on the expression of these two proteins during the development of the embryonic human kidney and imply that DAB1 may play a role in the development of mice kidneys by activating a variety of downstream pathways, including the Wnt signaling cascade [9].

The localization of inversin (cystoprotein) at the base of the primary cilium in renal tubular cells is considered to be obligatory in canonical Wnt signaling inhibition through cytosolic, but not membrane-bound DVL degradation, in the maintenance of normal tubular elongation and positioning, and its mutation subsequently provoked abnormal proliferation in tubular cells, resulting in CAKUT spectrum disease through the cystogenesis process [46,47]. Membrane-bound dishevelled switches on the non-canonical Wnt/PCP signaling. For convergent extension movements in gastrulating Xenopus laevis embryos and elongation of animal cap explants, both controlled by non-canonical Wnt signaling, inversin inhibition of canonical Wnt signaling is essential. Diversin, a switch molecule with structural similarity to inversin, treats renal cysts in zebrafish induced by inversin depletion, indicating that suppression of canonical Wnt signaling is required for successful renal development [48].

Therefore, we explored whether these markers’ expression and the staining pattern are altered in different mice kidney phases when comparing the healthy control group to the *yotari* mutant mice group. This investigation revealed a considerably lower percentage of *β*-catenin positive cells in the convoluted ducts and convoluted tubules of *yotari* mice at all observed embryogenic time points, indicating that *β*-catenin is needed for normal kidney morphogenesis and development. Our results demonstrated greater expression of *β*-catenin protein in the distal convoluted tubules of postnatal *yotari* kidneys compared to the control group, implying the switch from non-canonical to canonical Wnt/*β*-catenin signaling. Regular Wnt signaling pathway activation has been demonstrated to occur during tubulogenesis, but excessive canonical Wnt pathway activity in transplanted kidneys has been discovered to have a positive predictive value toward renal fibrosis [47,49]. Studies on AKI models following either ischemia or nephrotoxic insults have demonstrated that Wnt/*β*-catenin has a renoprotective effect, whereas tubule-specific ablation of *β*-catenin exacerbates damage [45]. However, prolonged and chronic stimulation of this signaling pathway may cause the onset and progression of CKD [50]. Taking all of this into account, *β*-catenin could be a potential therapeutic target against kidney fibrosis by reducing the hyperactivity of Wnt/*β*-catenin by natural compounds, particular inhibitors, or gene modification [29]. 

Wnt5a/b, which binds to both the canonical and non-canonical Wnt pathways, is a ligand for the seven-transmembrane receptor frizzled-5 and the tyrosine kinase orphan receptor 2. It is crucial for controlling developmental pathways throughout embryogenesis and the processes of oncogenesis. Global knockout of *Wnt5a* in mice resulted in pleiotropic but severe kidney phenotypes, including agenesis, fused kidney, hydronephrosis, and duplex kidney/ureter [51]. Wnt5a/b functions in planar cell polarity regulation in mice and disturbance in the PCP pathway has resulted in kidney cyst formation [27,52]. The study of Huang et al. showed Wnt5a morphants with disorganized pronephric cilia implying that Wnt5a may regulate proper kidney development by controlling ciliogenesis and PCP, which can be the aim of some further investigations [51]. Our results showed a significant increase of Wnt5a/b expression in proximal convoluted tubules of wild-type postnatal kidneys compared to *yotari*, which confirms that non-canonical Wnt/PCP signaling remains active after birth in a healthy kidney.

Wnt5a/b immunoexpression was observed in all structures during gestation, except the Ct at E13.5 in both mouse types, where *yotari* mice exhibit higher expression compared to wild-type mice. In the postnatal period, a significant increase in Wnt5a/b expression is observed in proximal convoluted tubules of wild-type kidneys compared to *yotari*, which confirms its role in nephrogenesis. The co-expression of Wnt5a/b and *β*-catenin was observed during nephrogenesis in metanephric mesenchyme of wild-type and *yotari* mice, but only occasionally in dct and pct during the postnatal development.

During normal kidney morphogenesis, the switch between the canonical and non-canonical Wnt-signaling pathway might be indicated by the protein immunoexpression pattern dynamics of acetylated α-tubulin, inversin, DVL-1, Wnt5a/b, and *β*-catenin throughout different kidney development phases. Their balance and expression in all investigated kidney structures imply their important role in normal kidney development. We suggest that their interchange determines transcription in the canonical Wnt pathway or order of cell migration and polarization during kidney development in a non-canonical signaling pathway. The critical finding of this study is that *β*-catenin and cytosolic DVL-1 levels are decreased in the MM of embryonic kidneys of *yotari* mice, compared to wild-type mice, while the levels of inversin and Wnt5a/b are increased. This implies that the switch from canonical to non-canonical Wnt signaling occurs earlier in *yotari* mice, possibly causing a premature end to nephrogenesis, which could be a contributing factor to their renal hypoplasia phenotype. Furthermore, *β*-catenin and cytosolic DVL-1 are upregulated in the postnatal kidneys of *yotari* mice, implying a switch from non-canonical to overactivated canonical Wnt signaling, which could be another contributing factor to renal hypoplasia in *yotari* mice or a possible compensatory mechanism. In contrast, healthy kidneys expressed inversin and Wnt5a/b in the postnatal period, thus maintaining non-canonical Wnt signaling. Therefore, disturbances of α-tubulin, inversin, DVL-1, Wnt5a/b, and *β*-catenin found in diseased *yotari* kidneys might be the underlying pathological mechanism and a result of the switch from non-canonical to canonical Wnt pathway in the developing and postnatal kidneys, resulting in CAKUT and impairment of kidney function, such as chronic kidney failure. 

## 5. Conclusions

The protein expression of acetylated α-tubulin, inversin, DVL-1, Wnt5a/b, and *β*-catenin in developing and early postnatal kidneys differs between *yotari Dab1^−/−^* and wild-type mice, indicating alterations in Wnt signaling. Wild-type kidneys display increased levels of *β*-catenin and cytosolic DVL-1 during the embryonic period, while increased inversin and Wnt5a/b levels characterize the postnatal period, indicating that a switch from canonical to non-canonical Wnt signaling is crucial for normal nephrogenesis. On the contrary, embryonic kidneys of *yotari* mice show decreased levels of *β*-catenin and cytosolic DVL-1 with increased levels of inversin and Wnt5a/b, implying an early switch from canonical to non-canonical Wnt signaling. The postnatal kidneys of *yotari* mice demonstrate a decreased inversin and Wnt5a/b levels and increased *β*-catenin and cytosolic DVL-1 levels, indicating a switch from non-canonical to overactivated canonical Wnt signaling, possibly resulting in a CAKUT spectrum disease.

## Figures and Tables

**Table 1:** Antibodies used for immunofluorescence.

Antibodies		Catalog Number	Host	Dilution	Source
Primary	Anti-acetyl-α-tubulin (Lys40) (6-11B-1)	12152S	Mouse	1:500	Cell Signaling Technology (CST), (Danvers, MA, USA)
	Anti-inversin	ab65187	Rabbit	1:100	Abcam (Cambridge, UK)
	Anti-dishevelled-1 (3F12)	sc-8025	Mouse	1:50	Santa Cruz Biotechnology (Dallas, TX, USA)
	Anti-Wnt5a/b (C27E8)	2530S	Rabbit	1:100	Cell Signaling Technology (CST), (Danvers, MA, USA)
	Anti-*β*-catenin (L54E2)	2677S	Mouse	1:200	Cell Signaling Technology (CST), (Danvers, MA, USA)
Secondary	Anti-Rabbit IgG, Alexa Fluor^®^ 488	711-545-152	Donkey	1:300	Jackson Immuno Research Laboratories, Inc., (Baltimore, PA, USA)
	Anti-Mouse IgG, Rhodamine Red™-X	715-295-151	Donkey	1:300	Jackson Immuno Research Laboratories, Inc., (Baltimore, PA, USA)

**Table 2:** Specific antibodies staining intensity in the kidneys of *yotari* and wild-type mice at embryonic days 13.5 (E13.5) and 15.5 (E15.5).

Embryonic Day (E)	Animal	Structure	Antibody				
			α-Tubulin	Inversin	DVL-1	Wnt5a/b	*β*-Catenin
E13.5	wild-type	Rv/G	−	++	+	−	+
		A/Cd	++	−	−/+	+	+++
		Ct	−/+	−	−	−	+
		MM	+	++	+	++	++
	*yotari*	Rv/G	−	++	+	+	+
		A/Cd	+++	+	−	−	+++
		Ct	+	−/+	−/+	−	+++
		MM	++	+++	++	++	++
E15.5	wild-type	Rv/G	−/+	++	++	+	−/+
		A/Cd	+++	−/+	−/+	−	++
		Ct	+	+	−	++	++
		MM	+	+++	+	+	+
	*yotari*	Rv/G	+	++	++	+	−
		A/Cd	+++	−	+	+	+++
		Ct	++	+	−/+	++	++
		MM	+	+++	++	+	+

+++ strong reactivity; ++ moderate reactivity; + mild reactivity; − no reactivity; Rv—renal vesicle; G—immature glomeruli; A—ampulla; Cd—collecting duct; Ct—convoluted tubule; MM—metanephric mesenchyme; E—day of embryonic development; Dishevelled (DVL-1).

**Table 3:** Staining intensity of specific antibodies in the kidneys of *yotari* and wild-type mice at postnatal days 4 (P4) and 14 (P14).

Postnatal Day (P)	Animal	Structure	Antibody				
			α-Tubulin	Inversin	DVL-1	Wnt5a/b	*β*-Catenin
P4	wild-type	G	+	−/+	+	−/+	−/+
		pct	+	−/+	−	++	+
		dct	++	+++	++	−	++
	*yotari*	G	−/+	+	+	−	−
		pct	+	−	−	+++	+
		dct	++	+++	++	−	+++
P14	wild-type	G	+	++	++	−/+	−
		pct	−	−	+	++	−/+
		dct	+	+	+	−	++
	*yotari*	G	−/+	++	++	+	+
		pct	+	−/+	+	+++	+
		dct	++	+	+	+	+++

+++ strong reactivity; ++ moderate reactivity; + mild reactivity; − no reactivity; G—glomeruli; pct—proximal convoluted tubules; dct—distal convoluted tubules; P—day of postnatal development; Dishevelled (DVL-1).

## Data Availability

All data and materials are available upon reasonable request.
